# Ryanodine receptor 1-related disorders: an historical perspective and proposal for a unified nomenclature

**DOI:** 10.1186/s13395-020-00243-4

**Published:** 2020-11-16

**Authors:** Tokunbor A. Lawal, Joshua J. Todd, Jessica W. Witherspoon, Carsten G. Bönnemann, James J. Dowling, Susan L. Hamilton, Katherine G. Meilleur, Robert T. Dirksen

**Affiliations:** 1 Tissue Injury Branch, National Institute of Nursing Research, National Institutes of Health, Bethesda, MD USA; 2 Neurogenetics Branch, National Institute of Neurological Disorders and Stroke, National Institutes of Health, Bethesda, MD USA; 3 Departments of Paediatrics and Molecular Genetics, Hospital for Sick Children and University of Toronto, Toronto, Canada; 4 Molecular Physiology & Biophysics, Baylor College of Medicine, Houston, TX USA; 5 Department of Pharmacology and Physiology, University of Rochester Medical Center, Rochester, NY USA

**Keywords:** Myopathy, Skeletal muscle, Clinical neurology, Ion channel defects, Neuromuscular disease, History

## Abstract

The *RYR1* gene, which encodes the sarcoplasmic reticulum calcium release channel or type 1 ryanodine receptor (RyR1) of skeletal muscle, was sequenced in 1988 and *RYR1* variations that impair calcium homeostasis and increase susceptibility to malignant hyperthermia were first identified in 1991. Since then, *RYR1*-related myopathies (*RYR1*-RM) have been described as rare, histopathologically and clinically heterogeneous, and slowly progressive neuromuscular disorders. *RYR1* variants can lead to dysfunctional RyR1-mediated calcium release, malignant hyperthermia susceptibility, elevated oxidative stress, deleterious post-translational modifications, and decreased RyR1 expression. *RYR1*-RM-affected individuals can present with delayed motor milestones, contractures, scoliosis, ophthalmoplegia, and respiratory insufficiency.

Historically, *RYR1*-RM-affected individuals were diagnosed based on morphologic features observed in muscle biopsies including central cores, cores and rods, central nuclei, fiber type disproportion, and multi-minicores. However, these histopathologic features are not always specific to *RYR1*-RM and often change over time. As additional phenotypes were associated with *RYR1* variations (including King-Denborough syndrome, exercise-induced rhabdomyolysis, lethal multiple pterygium syndrome, adult-onset distal myopathy, atypical periodic paralysis with or without myalgia, mild calf-predominant myopathy, and dusty core disease) the overlap among diagnostic categories is ever increasing. With the continuing emergence of new clinical subtypes along the *RYR1* disease spectrum and reports of adult-onset phenotypes, nuanced nomenclatures have been reported (*RYR1*- [related, related congenital, congenital] myopathies). In this narrative review, we provide historical highlights of *RYR1* research, accounts of the main diagnostic disease subtypes and propose *RYR1*-related disorders (*RYR1*-RD) as a unified nomenclature to describe this complex and evolving disease spectrum.

## Introduction

Congenital myopathies (CM), a term first coined by Victor Dubowitz [1], are a group of inherited, non-dystrophic neuromuscular disorders characterized by specific clinical features and skeletal muscle histopathology [2]. Pathogenic variations in the *RYR1* gene, a relatively large gene in the human genome, are the most common cause of CM and contribute to the clinical, histopathological, and genetic heterogeneity of CMs.

The *RYR1* gene (19q 13.2) encodes a calcium (Ca^2+^) release channel located in the terminal cisternae of the sarcoplasmic reticulum (SR) of skeletal muscle that is activated by Ca_V_1.1 voltage sensor proteins in the transverse tubule membrane during excitation-contraction (EC) coupling [3]. The purification of RyR1 Ca^2+^ release channels in 1988 [4] and the subsequent discovery in 1991 of pathogenic *RYR1* variants [5] led to the association of impaired Ca^2+^ homeostasis with malignant hyperthermia (MH) susceptibility (MIM # 145600) [6]. MH is a potentially fatal disorder of skeletal muscle Ca^2+^ regulation. MH episodes are triggered by exposure to certain volatile anesthetics (e.g., sevoflurane, desflurane) and depolarizing muscle relaxants (succinylcholine). MH episodes are characterized by uncontrolled muscle hypermetabolism, with a clinical presentation including hypercapnia, sinus tachycardia, masseter muscle rigidity, and hyperthermia [5, 7]. Guidelines from the European Malignant Hyperthermia Group (EMHG) and Malignant Hyperthermia Association of the United States (MHAUS) continue to inform the use of anesthetic drugs in individuals susceptible to MH [8, 9]. In certain cases, extreme heat conditions, fever, and/or exertion can result in symptoms that mimic MH episodes (enhanced heat response or EHS) [10–12]. Variations in the *RYR1* gene remain the leading cause of MH susceptibility; however, few MH-associated variations in other genes such as *CACNA1S* and *STAC3* have been described [13].

Functional analyses of RyR1 channels resulting from putative *RYR1* disease variants revealed multiple causative mechanisms including (1) increased sensitivity of RyR1 channels to activators (e.g., caffeine, halothane, Ca_V_1.1 voltage sensors) as observed in MH resulting in uncontrolled channel opening and Ca^2+^ release [14], (2) enhanced RyR1 Ca^2+^ leak [15], (3) reduction in RyR1 Ca^2+^ permeation leading to reduced Ca_v_1.1-mediated SR Ca^2+^ release, a process referred to as excitation-contraction uncoupling [16, 17], and (4) dramatic reduction in RyR1 channel expression [6]. In spite of these advances, many putative *RYR1* variants have not yet been directly tested/associated with Ca^2+^ release dysfunction, and thus, are classified as variants of uncertain significance (VUS) [18].

With a likely underestimated disease prevalence of 1:90,000 individuals [19], *RYR1-*RM is considered the most common form of non-dystrophic muscle disease in humans [20]. Of note, genetic variants resulting in MH susceptibility are more common, affecting approximately 1:3000–1:8500 [13], with a recent estimate from an exome analysis of a cohort of 870 individuals suggesting a prevalence of 1 in 400 [21]. *RYR1*-RM is inherited in both autosomal dominant and recessive manners and de novo cases have also been described. Clinical features suggestive of *RYR1*-RM are extensive, with mild to severe symptoms ranging from delayed motor milestones, proximal muscle weakness, hypotonia, and fatigue, to kyphoscoliosis, ophthalmoplegia, and moderate to severe respiratory insufficiency, which is more often apparent in recessive cases [22–24].

Historically, *RYR1*-RM subtypes were diagnosed and named based primarily on muscle biopsy histopathologic features such as central cores, cores with rods, central nuclei, fiber type disproportion, and multi-minicores. A number of earlier cases were described prior to *RYR1* gene identification and association with disease and, thus, may have been classified differently today. However, these histopathologic features are not unique to *RYR1*-RM, can be dynamic over time, may vary based on biopsy site, and may be absent when biopsy is performed at an early age [25, 26] or reflect a consequence of the gene dose (heterozygous = MH susceptibility versus homozygous = clinical myopathy) [27]. There are also several clinical and histopathologic similarities between the main *RYR1-*RM diagnostic categories of central core disease (CCD; MIM # 117000), multi-mini core disease (MmD; MIM #255320), core-rod myopathy (CRM), centronuclear myopathy (CNM), and congenital fiber-type disproportion (CFTD) [28, 29].

*RYR1*-related disorders can be viewed as occurring along a spectrum [30]. This spectrum includes *RYR1* variant-associated clinical phenotypes including King-Denborough syndrome, congenital neuromuscular disease with uniform type 1 fiber (CNMDU1), dusty core disease, rhabdomyolysis-myalgia syndrome, atypical periodic paralysis, and bleeding abnormalities [31–38]. Additionally, the spectrum of *RYR1*-related disorders has further expanded following reports of inherited, adult-onset phenotypes [39, 40].

Nuanced terminology is evident within the literature (including *RYR1*-related, *RYR1*-associated, *RYR1*-related congenital, *RYR1*-congenital) myopathies [28, 41–43]. The number of non-dystrophic neuromuscular disorders associated with *RYR1* genetic variations reflect the importance of the RyR1 protein in normal muscle function. Numerous informative reviews have been published on the *RYR1* disease spectrum [3, 13, 44–49]. This narrative review describes key historical milestones that led to the recognition of *RYR1-*related disorders as an overarching entity occurring along a complex clinical and histopathological spectrum. Here, we also summarize the current state of knowledge on the primary disease subtypes and propose a unified nomenclature that encompasses current and future phenotypes.

## Methods

As a preface, this is a historical narrative review. With input from subject-matter experts in *RYR1*-related disorders (including malignant hyperthermia), and information in landmark publications such as Magee and Shy [50] and Dubowitz and Pearse [51], the following search strategies were used for this narrative review: (1) computer search of databases for articles on congenital myopathies, and specifically, core myopathies; (2) review of congenital myopathy textbooks [52–55]; and (3) review of key articles of historical significance from reference lists of retrieved publications.

PubMed, ScienceDirect, and Scopus databases were searched without setting date limits or language exclusions. Chapters or sections from specialized neuromuscular textbooks or journals not annotated or available online were requested through the NIH Library. A timeline approach of landmark discoveries, starting from the earliest possible reports of congenital myopathies with related phenotypic features to that of *RYR1* subtypes was used as a framework for this historical perspective.

### Historical perspective of *RYR1-*related disorders

**Fig. 1:**
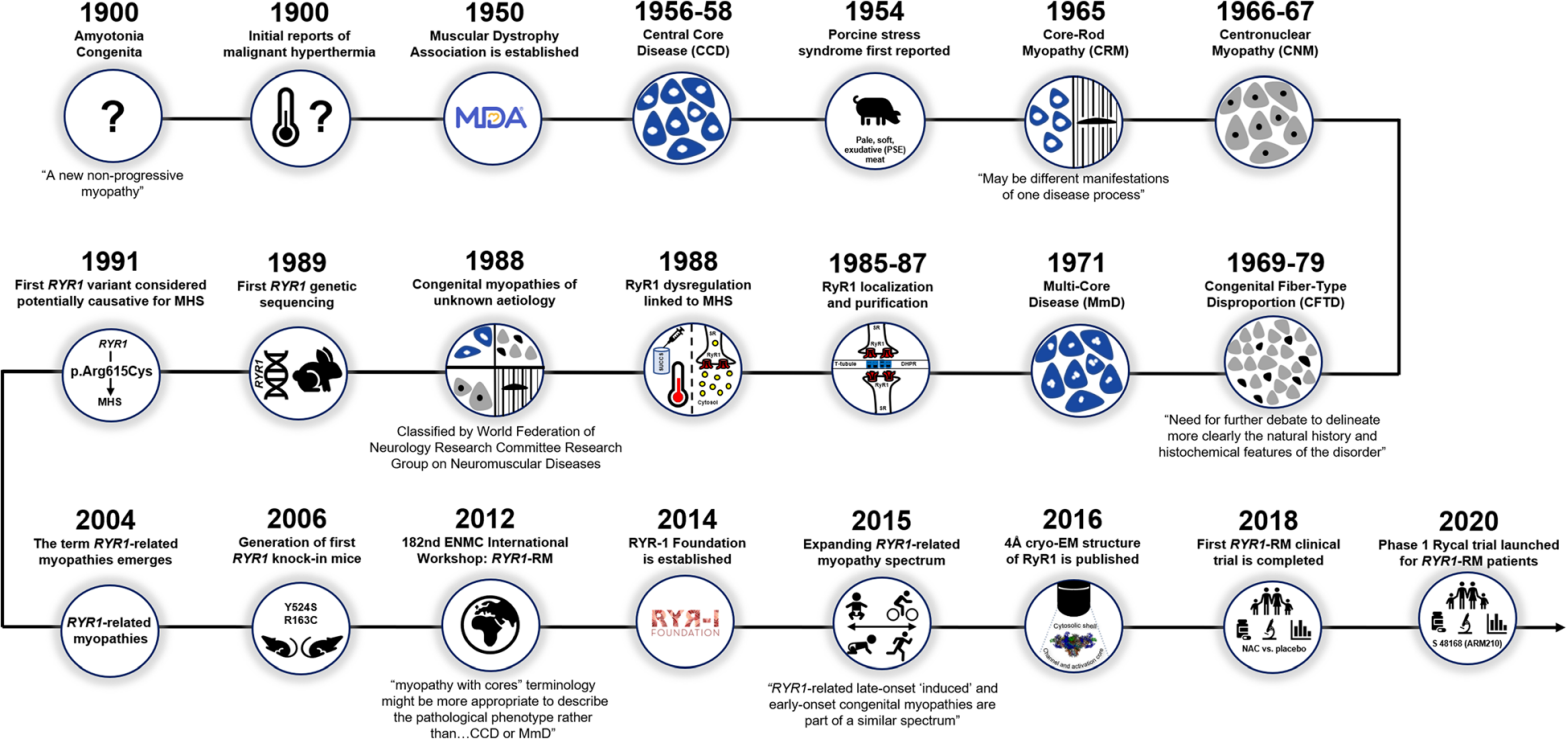
Timeline of significant discoveries/milestones in the evolution of *RYR1-RD*. 1900s to present: In the twentieth century, congenital myopathy diagnoses were based primarily on muscle biopsy histopathologic features. Advancements in next-generation sequencing enabled more precise identification of *RYR1*-related phenotypes. Solving of the RyR1 structure at near-atomic resolution provided valuable insight into RyR1 channel function and disease mechanisms. These advances paved the way for the first *RYR1*-RM clinical trial completed in 2018 and the Phase 1 Rycal trial which began enrolling participants in 2020

As with most historical reports, to our knowledge, none of the reported cases highlighted in this review prior to the advent of genetic sequencing have been resolved or unequivocally attributed to variations in the *RYR1* gene. Other genes currently associated with the histopathologic features of the main *RYR1*-related disorder subtypes are summarized in Table 1 and the historical timeline of their emergence is depicted in Fig. 1.

**Table 1:** Genes associated with main subtypes of *RYR1*-related myopathies

	Gene	Locus	Inheritance	Prevalence	Protein
**Central core disease**	*RYR1*	19q13	AD or AR	>90% [56]	Ryanodine receptor type 1
	*ACTA1*	1q42	AD	Rare	Skeletal α-actin
**Core-rod myopathy**	*RYR1*	19q13	AD or AR	Most common cause [2]	Ryanodine receptor type 1
	*NEB*	2q2	AR	Rare	Nebulin
	*KBTBD13*	15q25	AD	Rare	Kelch 13
**Centronuclear myopathy**	*RYR1*	19q13	AR	Most common cause of AR disease [28]	Ryanodine receptor type 1
	*DNM2*	19p13	AD	Most common cause of AD disease [2]	Dynamin-2
	*BIN1*	2q14	AR	Rare	Amphiphysin
	*CCDC78*	16p13.3	AD	Rare	Coiled-coil domain-containing protein 78
± cardiomyopathy	*TTN*	2q31	AR	Rare	Titin
Myotubular myopathy	*MTM1*	Xq28	XLR	Most common cause in severely affected males [57]	Myotubularin
**Congenital fiber-type disproportion**	*TPM3*	1q2	AD	25-50% [58]	α-tropomyosin
	*RYR1*	19q13	AR	~20% [59]	Ryanodine receptor type 1
	*ACTA1*	1q42	AD	Rare	Skeletal α-actin
	*TPM2*	9q13	AD	Rare	β-tropomyosin
	*SELENON*	1p36	AR	Rare	Selenoprotein N
± cardiomyopathy	*MYH7*	14q11	AD	Rare	Slow myosin heavy chain
	*HACD1*	10p12	AR	Rare	3-hydroxyacyl-CoA dehydratase 1
+ cardiomyopathy	Unidentified gene	Xp22.13 to Xq22.1	XLR	Rare	?
**Multi-minicore disease**	*SELENON*	1p36	AR	~50% [60]^a^	Selenoprotein N
	*RYR1*	19q13	AR	Second most common cause [61]	Ryanodine receptor type 1
± cardiomyopathy	*MYH7*	14q11	AR	Uncommon	Slow myosin heavy chain
+ cardiomyopathy	*ACTA1*	1q42	AD	Rare	Skeletal α-actin
+ cardiomyopathy	*DES*	2q35	?	Rare	Desmin
+ cardiomyopathy	*LMNA*	1q22	?	Rare	Lamin A/C

Adapted from Jungbluth, Sewry, & Muntoni, *The Congenital Myopathies*, in Rosenberg’s Molecular and Genetic Basis of Neurological and Psychiatric Diseases, Chapter 93, 5^th^ edition

*Abbreviations*: *AD* autosomal dominant, *AR* autosomal recessive, *XLR* X-linked recessive

^a^classic MmD phenotype. Only the most common genetic backgrounds and predominant modes of inheritance are indicated

#### Early twentieth century: amyotonia congenita

Most CMs in the early twentieth century were originally misclassified as amyotonia congenita, a condition initially described by neurologist Herman Oppenheim in 1900 [62] as general or local hypotonia of muscles in early infancy, with hyporeflexia, limb paralysis, absence of muscle wasting and familial involvement, and tendency to improve (Fig. 1).

In the early 1900s, the diagnosis of amyotonia congenita was given in cases involving sparing of the muscles supplied by the cranial nerves with muscular weakness and atony attributed to delayed muscle development. However, there were similarities in the clinical presentation of amyotonia congenita and infantile spinal muscular atrophy described by Werdnig and Hofmann in 1891 [63], a condition of progressive muscular flaccidity and weakness involving axial and proximal muscles due to loss of anterior horn cells in the spinal cord, as post-mortem records of some fatal cases of amyotonia congenita revealed lesions in the ventral horn cells and neuropathic atrophy [64]. These findings and the lack of clearly defined differentiating clinical features at the time resulted in an alternative erroneous hypothesis that the two conditions were variations of the same disease [65, 66].

#### Early twentieth century: post-operative heat stroke

Cases of hyperpyrexia associated with anesthesia during and immediately following surgical procedures were first reported in 1900 [67–71]. In 1919, written documents regarding the deaths of a mother and son anesthetized with chloroform and ether by G.A. Jones and E. Penny recounted muscle rigidity, violent and persistent respiratory muscle spasms, and rapid pulse [72]. However, the body temperature of the patients was not mentioned in this suggestive hereditary susceptibility to chloroform.

#### 1950s to 1960s: muscular degeneration

A disease termed muscular degeneration (MD) in pigs, manifested by discoloration of the skeletal musculature, was first reported in 1954 by J. Ludvigsen [73]. The altered musculature appeared gray or pale in color resembling that of chicken meat. MD was often fatal, especially when animals were exposed to exercise or stress. Prior to death the animals had dyspnea, cyanosis, circulatory insufficiency resulting from musculature vasoconstriction, and hyperthermia. Briskey and colleagues referred to this condition as pale, soft, exudative (PSE) tissue [74], and Kjolberg and colleagues referred to this condition as “white muscle disease” [75]. This alteration lowered the quality of the meat and therefore was a significant monetary loss to the meat processors and retailers. PSE was associated with predisposition to accelerated post-mortem glycolysis, and onset of rigor mortis at pH values below 5.9 and temperatures above 35 °C. PSE muscle ultrastructure showed disruption of sarcoplasmic components and protein filaments; however, the tissue appeared macroscopically normal. The economic implications of PSE meat generated a significant amount of research into porcine stress syndrome (PSS) [73, 76–78].

#### 1950s: central core disease

In 1956, Magee and Shy [50] investigated muscle biopsy findings from one large family with five affected members. Symptoms included non-progressive, infantile hypotonia with mostly proximal weakness. All biopsies from affected individuals showed similar appearances of larger than normal muscle fibers (up to 240μm in diameter), amorphous central cores in almost all fibers, and myofibrils that stained blue instead of purple with Gömöri trichrome stain. The myopathy resulting in this curious histopathological feature was later referred to as central core disease (CCD) by Greenfield and colleagues in 1958 [79], reflecting lack of oxidative enzyme activity in the amorphous cores due to mitochondrial depletion [51]. Additional histopathological features of CCD include increased internal and central nuclei, presence of rods, minimal to moderate endomysial fibrosis, increased fatty tissue and connective tissue infiltration, Z-line streaming, sarcomeric disorganization, fiber size variation, and predominance of type 1 fibers [79].

#### 1960s: malignant hyperthermia

In 1960, Michael Denborough and Roger Lovell described a case in which a young man nearly died following general anesthesia procedure with halothane for a compound fracture of the tibia and fibula [80]. There had been ten deaths attributable to general anesthesia, specifically ethyl chloride and ether, in the proband’s family. The pattern of inheritance was similar to an incomplete penetrant dominant gene or genes [81]. Available medical records showed that the course of events in the deceased family members had been similar, with convulsion and hyperthermia noted in two cases. This was the first breakthrough in recognizing MH as a heritable condition.

#### 1960s: core-rod myopathy

Shy and colleagues reported on a child with a non-progressive congenital myopathy and curved, thread-like structures at the periphery and center of affected muscle fibers on histopathological examination, and a clinical picture of a “floppy infant” [82]. Histopathologically, the lesion showed a spectrum of muscle fiber size ranging from 8 to 56 μm, with aggregates of palisading rod-like materials in the sub-sarcolemmal areas external to the myofibrils. The rods stained red on Gömöri trichrome stain, showed no polarization with white light or cross-banding, contained no formed inclusions such as mitochondria, and affected fibers had greater phosphorylase activity than unaffected fibers. These features reflected a new morphological muscle cell abnormality with highly organized protein-containing rod formations without cross-striations. This myopathic presentation was named nemaline myopathy because the structures could represent as rods or coils of thread-like structures.

Blurring the distinction between nemaline myopathy and CCD, a case of congenital myopathy with a combination of cores and rods was reported in one family in 1965 by Afifi et al. [83]. Skeletal muscle biopsies from the mother and daughter with autosomal dominant inheritance, congenital, non-progressive myopathy were examined. The daughter’s biopsy exhibited CCD features, while the mother’s biopsy exhibited characteristics of both CCD and nemaline myopathy. The authors suggested that CCD and nemaline myopathy could be manifestations of one disease process, with various means of expression, because it would be most unusual to find two unrelated rare diseases with similar clinical features and inheritance patterns in the same individual.

#### 1960s: centronuclear myopathy

An undescribed, slowly progressive myopathy characterized by the presence of central nuclei in about 85% of muscle fibers was referred to as myotubular myopathy (MTM) by Spiro and colleagues in 1966 [84] and familial centronuclear myopathy (CNM) by Sher and colleagues in 1967 [85]. CNM subtypes were unified histopathologically by small myofibers containing hyperchromatic central nuclei with wrinkled or serrated borders in > 25% of muscle fibers, and rows or aggregates of up to thirty nuclei on hematoxylin and eosin stain. The central nuclei are usually surrounded by an unstained space.

#### 1960s to 1970s: congenital fiber-type disproportion

Following a series of detailed histological studies of variations in muscle fiber types in neuromuscular diseases [86], Michael Brooke coined the term congenital fiber-type disproportion (CFTD) to describe consistently smaller type 1 muscle fibers than type 2 fibers in fourteen patients, with fiber size disproportion (FSD) greater than 12% in the absence of any other histological abnormality [87]. CFTD was initially considered to be a non-specific feature that preceded the development of more specific histologic features, as other CMs presented with FSD and corresponding clinical manifestations [88].

#### 1970s: multi-minicore disease

Multiple small core-like structures on skeletal muscle biopsy in two siblings with CM, termed multicore disease, were first reported by Engel and colleagues in 1971 [89]. Different from the central cores of CCD, these variable, indistinct multiple mini cores of areas with decreased oxidative activity extend only a short distance along the length of the muscle fiber, with some larger mini cores stretching across the fiber width.

#### 1980s to 1990s: RyR1 localization and malignant hyperthermia linkage

The characterization of RyR1 ryanodine binding [90, 91], physiology of PSS and identification of porcine MH (*hal*) locus [92–94], channel activity [95, 96], biochemical purification [97, 98], cloning [99–101], and the initial classification of CCD, MmD, and CFTD as “congenital myopathies of unknown aetiology” [102] all took place in the mid-late 1980s. Linkage analysis found MH sensitivity to co-segregate with chromosomal markers in the *RYR1* gene with a lod score of 4.20, consistent with MH being caused by mutations in the *RYR1* gene in 1990 [103]. These studies naturally prompted the search for specific *RYR1* pathogenic variants and led to identification of the first MH causative variant in 1991 (c.1843C>T; p.Cys615Arg) [5, 104] and then subsequently for CCD in 1993 [105].

#### 2004: imaging of intramuscular fatty infiltration as a diagnostic tool

The wide range of phenotypes associated with *RYR1* variants, the large size of *RYR1*, and complex histopathological overlap across different CMs due to variants in other genes encoding sarcolemmal and sarcotubular proteins [29] present challenges in confirmatory diagnosis. *RYR1*-RM-affected individuals show a consistent pattern of relative sparing of rectus femoris, adductor longus, hamstring muscles, the medial head of the gastrocnemius (except in calf-predominant myopathy) [106], and muscles of the anterior compartment of leg [107]. In 2004, Jungbluth and colleagues used magnetic resonance imaging (MRI) to classify distinct patterns of selective muscle fatty infiltration using in congenital myopathies associated with *RYR1* causative variations [107]. As a result, muscle MRI is now used to supplement clinical assessment and genetic testing for the diagnosis of patients with variable histopathology. The term “*RYR1*-related congenital myopathies” was used to describe the distinct pattern of muscle involvement reported.

#### 2006: generation of first knock-in mouse models of RYR1-related myopathies (RYR1-RM)

Creation of a knock-in mouse heterozygous for the Y524S variation (equivalent to Y522S in humans) by Chelu et al. [108] was the first to represent a murine model of MH. These mice experienced whole body contractions and elevated core temperatures in response to isoflurane exposure or heat stress without uncompensated SR calcium leak or store depletion. In the same year, Yang and colleagues created another valid MH susceptible *RYR1* knock-in mouse heterozygous for R163C [109]. The R163C heterozygous mouse SR membranes have a twofold higher affinity (Kd = 35.4 nm) for [H]ryanodine binding compared with wild type.

#### 2011–2012: a spectrum of RYR1-related myopathies (RYR1-RM)

As histological phenotypes associated with *RYR1* variations can continually evolve over time in the same patient or vary in individuals with the same variant, terminologies that include these features might not be appropriate for specific designations [110]. The expanding histopathologic (from normal biopsy findings in MH susceptible individuals, histology typical of CCD, MmD, CNM, and CFTD, to cores in MH susceptible individuals, type 1 fiber predominance, and mixed histopathology of cores and rods) and emerging *RYR1*-RM phenotypes were the focus of the European Neuromuscular Centre (ENMC) international workshops in 2011 and 2016 [27, 111]. These consensus-building workshops delineated *RYR1*-RM as a spectrum of clinical and pathologic phenotypes, with histolopathologic features ranging from subtle abnormalities such as increased internalized nuclei to prominent and extensive structural cores. The wide range of *RYR1*-RM histopathological features, with mild myopathic changes and irregular oxidative staining in the late-onset phenotypes and more frequent detection of cores in the congenital myopathies, is suggestive that both are part of a similar spectrum [30]. With reports of adult-onset phenotypes [39, 40] resulting from increased availability of diagnostic exome and whole gene *RYR1* sequencing [112], the full spectrum extends beyond birth and early childhood. A confounding factor in assessment of late-onset cases is advancing age, as aged muscle exhibits varying degrees of increased intramuscular fat content on MRI, muscle fiber atrophy and loss, tubular aggregates, and gradual increases in ragged red fibers and cytochrome c oxidase-negative fibers [113].

#### 2015–2016: advances in RyR1 structure-function

The molecular architecture of RyR1, solved using a combination of cryo-electron microscopy (cryo-EM) and x-ray crystallography of soluble subdomains, reveals a homotetrameric complex with a molecular weight of 2.25 million Da, consisting of four protomers (~ 565 kDa each) that interact with other regulatory proteins and ligands [114, 115]. Advancements in cryo-EM and direct electron detector (DED) technology enabled the most comprehensive 3D reconstruction of this 5038-amino acid structure to date. Indeed, between 2015 and 2016, a series of studies reported RyR1 structure at near-atomic resolution, ranging from 3.6 to 4.8 Å [114, 116–118]. The cryo-EM reconstructions revealed binding sites for channel agonists and antagonists, as well as the structural basis of channel gating and ligand-dependent activation. Cytoplasmic interacting proteins (e.g., FKBP12 or calstabin1 and calmodulin or CaM) and SR proteins (e.g., triadin and junctin) bind and regulate RyR1 channel activity [119]. In the absence of channel activators (Ca^2+^, ATP, caffeine), the RyR1 core is rigid and remains in a closed state. In contrast, channel activator binding at different sites on the RyR1 C-terminal domain (CTD) serves to increase transition to and stability of the channel open state [118].

#### 2018: completion of the first RYR1-RM natural history study and clinical trial

There is no approved treatment for *RYR1*-RM. Symptom management is primarily supportive, with precautions taken when MH risk is either known or not ascertained. There have been anecdotal reports of positive responses to pyridostigmine, an acetylcholinesterase inhibitor, improving fatigue and energy level [43], and salbutamol, a beta agonist that improves muscle strength and motor function through mechanisms that are not completely understood [120]. Development of patient registries for clinical trial recruitment and funding support from patient advocacy groups such as the Muscular Dystrophy Association (MDA), the RYR-1 Foundation, CureCMD, and congenital muscle disease international registry (CMDIR) have been instrumental in supporting therapeutic development for neuromuscular diseases. The first clinical trial for *RYR1*-RM was conducted in ambulatory individuals and included a 6-month lead-in natural history phase followed by 6-month intervention with the antioxidant *N*-acetylcysteine (NAC) [121] (NCT02362425). The rationale for this clinical trial was evidence that NAC rescued elevated oxidative stress and decreased myopathy in murine [122] and zebrafish model systems [123, 124]. All participants exhibited elevated oxidative stress as determined by urine 15-F2t-isoprostane concentration and decreased physical endurance. However, oral treatment with NAC did not impact either outcome [121]. Other therapeutic research opportunities investigating the efficacy of modulators of calcium release from the SR are underway globally. For *RYR1*-RM-affected individuals, a phase 1 clinical trial testing safety of the RyR stabilizing Rycal molecule S48168 (ARM210) is underway (NCT04141670). Moreover, several drugs already approved for other indications are in the clinical trial pipeline for potential re-purposing, and novel compounds, identified by high-throughput screening, are being tested in pre-clinical studies [125–127]. The recent generation of murine model systems that more closely depict *RYR1*-RM clinical phenotypes [128, 129] and greater understanding of RyR1 structure-function [130] will play a crucial role in identifying *RYR1*-RM-affected individuals who could benefit most from specific therapeutics. However, the heterogenous nature of the disease suggests that a single treatment is unlikely to be universally efficacious.

### Current state of knowledge and differential diagnoses

#### Central core disease (CCD)

Cores associated with *RYR1* variations may be structured or unstructured based on ATPase activity levels (positive, structured; absent, unstructured) and myofibrillar disruption. These cores are typically seen in type I fibers with significant fibro-adipose infiltration [131]. *RYR1*-associated CCD is predominantly an autosomal dominant condition. In autosomal dominant and de novo cases of CCD, *RYR1* variations predominantly affect the RyR1 C-terminal region [56]. *RYR1* variations resulting in CCD and MH susceptibility were initially reported to primarily localize in three “hot spot” regions (or domains): domain 1 (N-terminal residues 1–614), domain 2 (central residues 2163–2458), and domain 3 (C-terminal pore/transmembrane residues 4136–4973) [3, 56]. However, more recent information indicates that dominant *RYR1* variations can span the entire length of the gene [111].

The clinical features of CCD are variable, and approximately one third of individuals with central cores do not exhibit an overt clinical phenotype [132]. Clinical characteristics of autosomal dominant CCD include hypotonia, developmental motor delay, proximal weakness, myalgia, and orthopedic complications such as scoliosis and hip girdle dislocation. Cardiac, bulbar, and moderate to severe respiratory involvement are rare [6]. Infantile and early childhood presentation with proximal weakness in the hip girdle is typical [55] and, although typically stable over time, slow disease progression has been reported later in life [133]. Recessive cases of CCD are rare but can present with more severe features including, arthrogryposis, respiratory distress and fetal akinesia [29, 134]. CCD is considered allelic to MH susceptibility, which is also predominantly associated with a dominant mode of inheritance [53]. Given this genetic connection, a subset of individuals with CCD diagnoses are MH susceptible, and a subset of MH susceptible individuals present with cores on their muscle biopsies [13]. RyR1 channels with MH- or CCD-associated variants show higher activity and sensitivity to activation than wildtype channels, which ultimately leads to increases in resting Ca^2+^ concentration [135, 136]. Clinical and histopathologic findings in *RYR1*-RM-affected individuals are widely variable and often also present in other congenital myopathies. Greater than 90% of cases with typical CCD clinical manifestations and histopathology result from *RYR1* variations [56]. However, structures similar to cores are also observed in *ACTA1*-associated myopathy [137]. Target fibers may be confused with central cores as they are characterized by absence of oxidative enzyme activity, paucity of mitochondria, and disorganized myofibrils in the center, surrounded by a rim of more intense than normal activity on immunohistochemical staining of muscle biopsy [138, 139].

#### Core-rod myopathy

Although histologically distinct, the presence of cores and rods in the same muscle biopsy examination has been described in other cases with *RYR1*-RM [110, 140]. *RYR1* variations are the most common cause of core-rod myopathy and both dominant and recessive forms have been described [110, 141]. Variations in the *NEB* [142] and *KBTBD13* [143] genes have also been implicated in core-rod myopathy, with *KBTBD13*-related forms associated with slow muscle movement and proximal weakness [116, 144]. Other genes associated with nemaline myopathy include *ACTA1, TPM3, TPM2, TNNT1,* and *CFL2*. Kondo and colleagues reported on a patient diagnosed with severe congenital nemaline myopathy and compound heterozygous variations in *RYR1* [145]. Clinical manifestations of this patient included fetal akinesia, severe generalized hypotonia, narrow face with facial muscle weakness, persistent ophthalmoplegia, frog-leg posture, poor-anti-gravity limb movements, respiratory insufficiency, and an improving clinical course [145]. Histologically, nemaline bodies (observed as numerous small rods) were observed in the cytoplasm but not in the nuclei, and small type 1 fibers without central nuclei, fiber degeneration, or cellular infiltration were noted. No central cores or minicores were observed in this patient. The absence of cores and the presence of nemaline rods may differentiate this case from previous reports of recessive *RYR1* cases but could also be reflective of delay in core presentation on muscle histology in young patients [146, 147]. Identification of cores or minicores on later biopsies would therefore classify this case as a recessive core-rod myopathy, which is most commonly associated with *RYR1* variations [140].

#### Centronuclear myopathy (CNM)

Mitochondrial oxidative enzyme activity is either concentrated or absent in the centrally nucleated fibers (core-like areas) [148]. The case reported by Spiro and colleagues could be attributable to variation(s) in one of the genes associated with CNM. Of the nine genes currently associated with CNM (*RYR1, MTM1, DNM2, BIN1, TTN, MTMR14, SPEG, CCDC78,* and *CACNA1S*), *RYR1* variants are the most common cause of autosomal recessive CNM [28]. CNM symptoms predominantly affect skeletal muscles. Clinical features of *RYR1*-related CNM include extremity muscle weakness (typically severe), foot abnormalities, scoliosis, ophthalmoparesis, and mild to severe respiratory involvement [146]. In individuals with CNM, disease severity is extremely variable with the majority of cases exhibiting compound heterozygous changes. Variations in the *MTM1* gene should be investigated first in severely affected males, with analysis of cDNA from muscle tissue recommended if a variation is not identified on genomic DNA [57]. Females with *MTM1*-related CNM may present with necklace fibers as a histologic marker [149]. *DNM2* variations should be investigated first if there is a clear autosomal dominant family history or de novo variant [2, 150].

#### Congenital fiber-type disproportion (CFTD)

As Brooke’s definition of > 12% FSD rendered CFTD a non-specific diagnosis, FSD greater than 35–55% with clinical features consistent with CM is currently used as a diagnostic criterion [151]. Both dominant and recessive cases of *RYR1*-related CFTD have been reported [59]. Clinical features reported in CFTD patients include hypotonia, respiratory failure, non-progressive muscle weakness, joint contractures, myopathic facies, ophthalmoparesis, feeding difficulties, and skeletal deformities [59, 152]. It is important to note that many patients initially diagnosed with CFTD develop rods, cores, and central nuclei over time, leading to specific diagnoses that supersede the initial CFTD diagnosis [2, 151]. With an autosomal dominant mode of inheritance, *TPM3* is the most common genetic cause of CFTD (25–50% of cases) [58]. *RYR1*-related CFTD is an autosomal recessive disease accounting for about 20% of CFTD cases [59]. *ACTA1* and *TPM2* are also uncommon causes of CFTD [153, 154].

#### Multi-minicore disease (MmD)

The variability associated with the appearance of cores on muscle biopsy led to subsequent reports of multicore disease with various nomenclatures (minicore myopathy, multicore myopathy, multi-minicore myopathy) until a working group of experts agreed on a designation of multi-minicore disease (MmD) [61]. Multi-minicores may affect both type 1 and type 2 muscle fibers and show depletion or absence of mitochondria on electron microscopy, with variable degrees of myofibrillar disorganization, abnormal Z-band material, and regions of sarcomeric disruption. Increased internal nuclei, fibers with slow myosin (type 1 predominance on histochemical stains), prominent connective tissue and adipose tissue in the absence of numerous fibers with developmental myosin, necrosis or endomysial fibrosis are also noted in affected muscles [141, 155]. *RYR1*-related MmD is inherited in an autosomal recessive pattern with highly variable clinical features [156]. Typical features include hypotonia in infancy, axial muscle weakness, hip girdle weakness, ophthalmoplegia, distal joint laxity, progressive scoliosis, and moderate respiratory/bulbar involvement. Four homogeneous MmD groups have been identified: (1) the classic form, marked by predominantly axial muscle weakness, especially neck flexors, scoliosis, respiratory insufficiency, and limb joint hyperlaxity; (2) the ophthalmoplegia form, with generalized muscle involvement and severe facial weakness; (3) an early-onset form with arthrogryposis; and (4) a slowly progressive form with hand amyotrophy [157]. Other genes associated with MmD include *SEPN1* [60], *MYH7* [158], and *TTN* [159]. Pathogenic variants in *SEPN1* and *RYR1* are responsible for approximately 50% of all cases and *SEPN1* variations comprising ~ 75% of the classic form of MmD [60]. *RYR1* variations are mostly associated with both the moderate form of MmD with hand involvement and the ophthalmoplegic form [156, 160], both of which typically present as milder than classic *SEPN1*-associated MmD. Multi-minicores associated with *SEPN1* variations are typically smaller in size than those observed in recessive *RYR1*-related cases [131] and are accompanied by non-specific myopathic changes such as CFTD and Mallory body-like inclusions [161, 162]. Malignant hyperthermia and ophthalmoplegia are not usually noted in *SEPN1*-related myopathies [163]. On MRI, there is prominent involvement of the sartorius muscle in the thigh, and in more severe cases, the pattern of thigh involvement show similarity with that seen in *RYR1*-RM [164]. *MYH7*-, *DES*-, *LMNA*- and *TTN*-related core myopathies are recessive, progressive, and often present with severe cardiomyopathy that can be independent of respiratory insufficiency [158, 159, 165, 166]. Joint hypermobility, although more often a sign of connective tissue disorders, can be a prominent clinical feature of *RYR1*-related core myopathies [167]. Additionally, the presence of a large number of muscle fibers (up to 50%) with internal or central nuclei has been reported as part of the *RYR1*-related core myopathy spectrum [168]. Minicores are also non-specific features of congenital muscular dystrophies, dystrophinopathies, neuropathies, short-chain acyl-COA dehydrogenase deficiency, Marfan syndrome, and cardiomyopathies [2, 160]. Nemaline bodies with core-like areas have been described in dominant *ACTA1*-related myopathy [169] and in a family with recessive *CFL2* variations [170]. Multiple core-like areas per fiber were reported in type-1 fibers in *DOK7*-associated congenital myasthenic syndromes [171] and *RYR1*-associated atypical periodic paralysis [35]. “Moth-eaten” fibers described in certain muscular dystrophies can also be considered core-like lesions [172]. However, these small core-like lesions are often not confirmed with electron microscopy compared with multiple minicores. Variations in *MYH7* and *TTN* should be considered if there is an associated cardiomyopathy.

### Proposal for a unified nomenclature

**Fig. 2:**
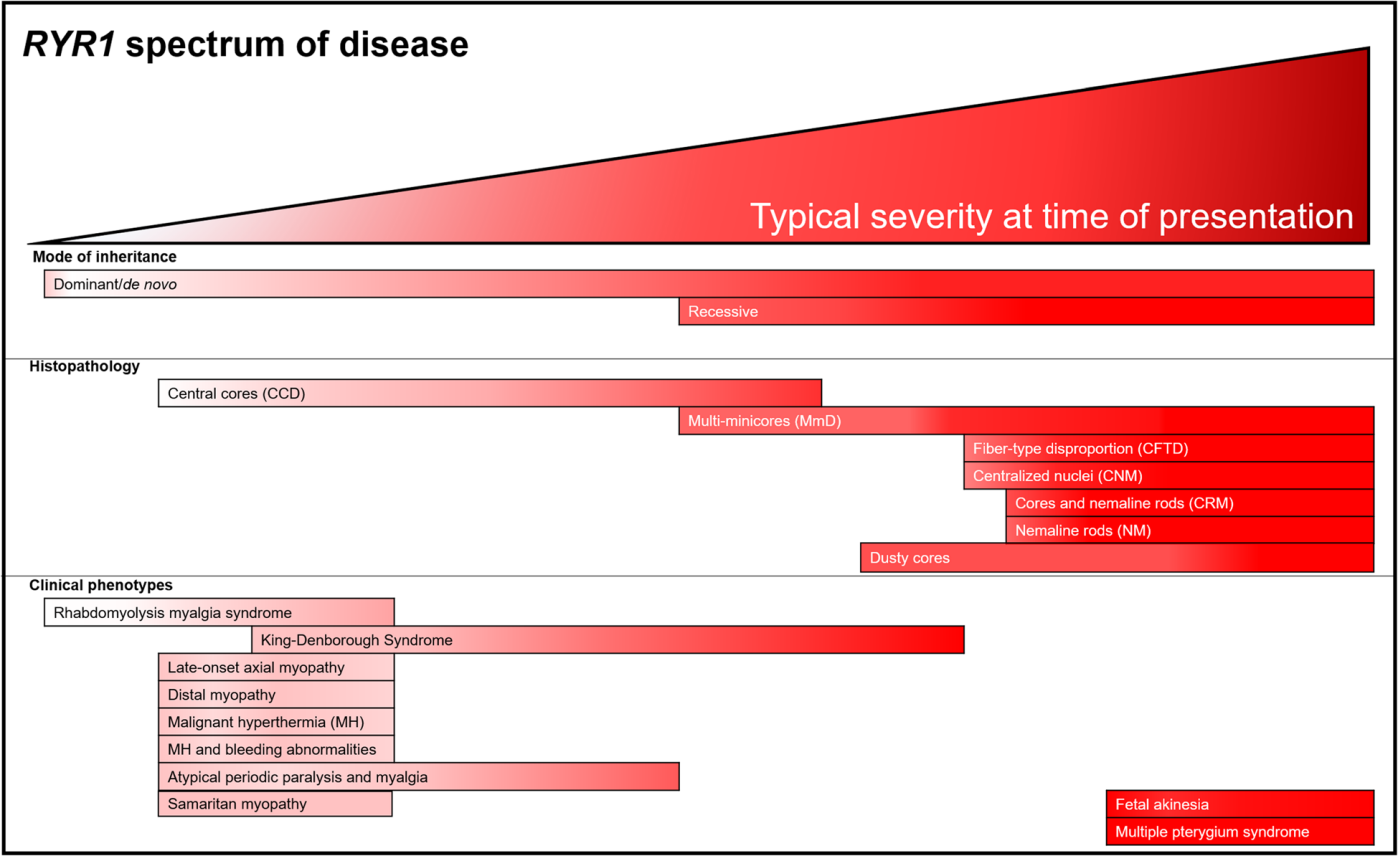
Overview of the *RYR1* disease spectrum. At time of presentation, clinical severity can vary according to mode of inheritance (dominant, de novo, recessive), histopathologic features, and phenotypes ranging from severe neonatal onset to mild non-progressive muscle weakness. Recessive cases are typically more severe than dominant cases. The majority of histopathological features are associated with more severe clinical phenotypes, though this may not hold true for the core myopathies. Emerging clinical phenotypes associated with *RYR1* variations also vary in severity

The similarity in muscle histopathology and clinical symptomatology across *RYR1*-RM subtypes, currently viewed as nosocologically distinct entities, reflects a wide range of manifestations resulting from defects in the same gene. Specifically, patient severity occurs on a spectrum and is often evaluated in the context of mode of inheritance, histopathology, and clinical phenotype (Fig. 2). A combination of factors has made understanding and describing muscle disorders associated with *RYR1* variants confusing for health care providers, researchers, and patients/families. Moving forward, we therefore propose the term “*RYR1*-related disorders (or *RYR1*-RD)” as a single nomenclature to unify this complex myopathic/non-myopathic and congenital/non-congenital spectrum with the goal of minimizing the following known issues.

#### Dynamic and overlapping histopathology

Historical classification and diagnosis of newly emerging *RYR1*-RD based on clinico-histopathologic features are complicated because findings often overlap with previously identified phenotypes. The histopathologic features used to diagnose *RYR1*-RD are variable over time, and therefore, subject to inconclusive findings. The degree of pathological changes may vary by biopsy site or age of the individual when the biopsy was obtained. Very young patients may not present definitively with cores until later in life [173] and some CCD cases with MH susceptibility may not present with cores [131]. Clinical features of dominant CCD typically present on the milder end of the phenotypic spectrum including hypotonia, muscle weakness, and skeletal abnormalities in the absence of cardiac involvement [131]. However, the rare cases of recessive CCD that often exhibit a more severe clinical presentation [174, 175] do not always correlate with histopathologic findings [42].

#### Delayed diagnosis

Diagnosis is delayed and difficult when nosocologically defining histopathologic features are absent, and in cases with dual morphologic presentations such as core-rod myopathy [131]. Also, the absence of diagnostic morphologic features on muscle biopsy does not exclude a likely pathogenic variation in *RYR1* [61]. With nearly 700 *RYR1* variations identified to date [176], availability and access to exome sequencing and genetic testing of the entire *RYR1* gene can be credited for early and faster diagnosis [177], and expansion of the *RYR1* disease spectrum [178]. A recent *RYR1*-RM natural history study [179] revealed that affected individuals born before the advent of next-generation sequencing (2004) were typically diagnosed as adults, while those born after 2004 were generally diagnosed in early childhood. Additionally, homozygous or compound heterozygous probands often exhibit a profound myopathic phenotype, while heterozygous probands might only experience a triggered phenotype. A genetics-first diagnostic approach is rapidly becoming the standard for confirmation of disorders with known genetic etiology [112, 180]. Nevertheless, the limited sensitivity of next-generation sequencing, requirement for multiple testing in some cases, and interpretation of large numbers of identified VUS in a relatively large gene such as *RYR1* still requires a robust knowledge of the suggestive features associated with each variant. Although complete reliance on histopathologically defined entities such as central cores, multi-minicores and central nuclei is arguably outdated, these muscle biopsy findings remain valuable as diagnostic indicators of disease subclassification that are not otherwise feasible from genetic findings alone. *RYR1*-RD affected individuals exhibit specific and valuable initial patterns of clinical and histopathologic presentations that can facilitate early diagnosis. As the possibility of an inherited process is not always apparent [181], clinical, histopathologic, and genetic information still need to be evaluated together in order to reduce the diagnostic odyssey of patients and their families [182].

#### Adult-onset phenotypes

*RYR1*-RD are typically considered primarily early childhood-onset conditions with proximal or generalized muscle weakness. Recent identification of adult-onset subtypes reflects a departure from the long-held definition of this group as strictly “congenital myopathies” [39, 40]. Considered a late neuromuscular manifestation of MH-related *RYR1* variations, late-onset axial myopathy, exertional rhabdomyolysis, and periodic paralysis present throughout the lifespan. Late-onset *RYR1*-RD present significant diagnostic and genetic counseling challenges, as well as implications for proper anesthetic management of patients and their family members. True adult-onset cases are difficult to establish as the affected individual could have either tolerated or been in denial of mild symptoms experienced during childhood. *RYR1*-RD phenotypes are still predominantly early childhood onset, and excluding the “congenital” descriptor does not detract from their classification as CM. However, awareness of these adult-onset subtypes allows: (a) clinicians to consider *RYR1*-RD in the differential diagnostic process across all ages even in neuromuscular disease cases without prior muscle biopsy findings [39], (b) inclusion of older adults in clinical trials testing novel therapies, and (c) collection of more robust natural history data on *RYR1*-RD across the lifespan [37].

#### Shared calcium dysregulation

*RYR1*-RD result from varying disease pathomechanisms that collectively share alterations in a common pathway—intracellular calcium dysregulation resulting from primary RyR1 dysfunction (e.g., reduced RyR1 expression, leaky RyR1 channels, impaired RyR1 interdomain interactions, enhanced sensitivity to modulators, impaired excitation-contraction coupling) [6, 22]. Alterations in calcium homeostasis can also lead to secondary cellular dysfunction including increased oxidative/nitrosative stress, altered post-translational modifications, mitochondrial damage, and disrupted protein-protein/ligand interactions [3, 122, 183]. These downstream effects further drive myopathy and enhance heat responsiveness due to a feed-forward loop [11]. However, RyR1- or cellular/mitochondrial-based calcium dysregulation such as increased mitochondrial calcium uptake, production of damaging reactive oxygen species [3, 122, 183] and upregulation of endoplasmic reticulum stress/unfolded protein response [184], may be *RYR1* variant dependent.

## Discussion

The complex nature of the RyR1 protein, coupled with the expanding and overlapping disease spectrum of *RYR1*-RD, presents a timely opportunity to consider a unified nomenclature and classification system for this heterogeneous group of disorders. Here, we propose the use of “*RYR1*-related disorders (*RYR1*-RD)” as a single nomenclature to unify this complex myopathic/non-myopathic and congenital/non-congenital spectrum.

Any acceptable change in nomenclature will require a careful, widely discussed and expansive evaluation by experts in the field of neuromuscular disorders. A comprehensive classification system would both incorporate the different disease pathomechanisms associated with *RYR1*-RD and identify potential consequences that may not be immediately apparent. A unified nomenclature is needed for multiple reasons, but most importantly to facilitate unambiguous communication about related conditions among clinicians, researchers, patients, and the lay public. A relevant example in the neuromuscular disease field is the consensus naming and classification of multi-minicore disease (MmD) by a panel of experts [157]. Following the first reports of MmD [89], numerous cases with variable clinical expression and morphological lesions were reported. This led to multiple histologic descriptors including multicore disease, focal loss of cross-striations, minicore myopathy, myopathy with multiple minicore, or pleocore disease to describe the same disease subtype [60]. In comparison to relatively more homogeneous disorders such as *SEPN1*-RM [60] and *COL6*-RM [185] where gene-based nomenclatures were introduced, there is an inherent possibility that a single nomenclature for such a clinically and histopathologically heterogeneous group of disorders may be seen or interpreted as having the same pathomechanisms and genotype-phenotype correlations.

*RYR1*-RD subtypes also present clear clinicopathological and mode of inheritance differences. For example, there are no reported pedigrees with unequivocal evidence for multigenerational dominant inheritance of *RYR1*-related CFTD and CNM, both overwhelmingly associated with recessive *RYR1* variations. Individuals with core-rod myopathy present with excess ryanodine receptor levels in the cores [140], while MmD and recessive forms of CCD exhibit a marked reduction in RyR1 protein expression [186]. Although type 1 fiber predominance and hypotrophy are observed in most cases, type 1 fiber uniformity without structural changes (cores and rods) is found in over 99% of the type 1 muscle fibers in certain cases with C-terminal *RYR1* variants [33]. From a clinical perspective, extraocular muscle involvement is almost exclusively associated with recessive forms of *RYR1*-RD, whereas enhanced malignant hyperthermia susceptibility is more commonly seen with dominant rather than recessive *RYR1* variations [187].

Additionally, some degree of dysfunction in calcium homeostasis and/or E-C coupling is a shared pathomechanism among *RYR1*-RD. The effects of specific *RYR1* variants on these mechanisms depend on mode of inheritance and location on the gene [179]. Therapeutic development for *RYR1*-RD aiming to prevent RyR1 calcium leak may not be beneficial and could potentially be detrimental, in the context of dominant variants that reduce RyR1 calcium conductance or recessive variants that lead to a dramatic reduction in RyR1 expression. The different pathomechanisms caused by *RYR1* variations (e.g., hypersensitivity, enhanced calcium leak, E-C uncoupling, decreased RyR1 channel expression) [6] and the prospect of distinct therapeutic approaches needed to combat these different underlying disease mechanisms are seemingly at odds with the concept of a unified nomenclature. Finally, unified nomenclature may have unintended negative implications for research funding opportunities from patient advocacy organizations focused on specific congenital myopathies and may present challenges in formulation of treatment, biomarkers, and/or clinical outcome measures across subtypes with pathomechanisms that affect varying stages of cellular functions. This could slow the pace of drug development and treatment approval by regulatory agencies.

**Fig. 3:**
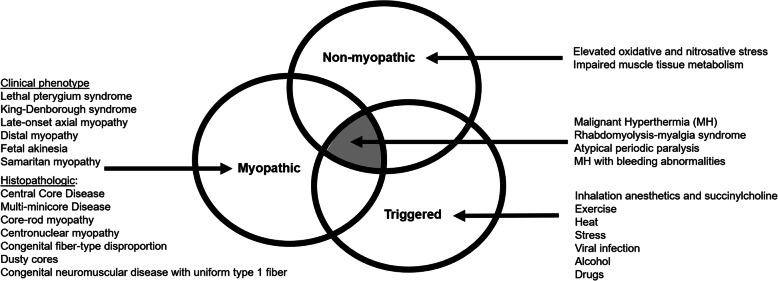
Distinct and overlapping features of the *RYR1*-RD spectrum. Individuals with *RYR1*-RD present with both myopathic and non-myopathic features. Histopathology can overlap among the different subtypes. Additionally, some phenotypes manifest following exposure to pharmacologic, physiologic, or environmental triggers. A classification system based on three distinct and overlapping categories (myopathic, non-myopathic, and triggered) accommodates current and most future subtypes of *RYR1*-RD

In our opinion, a unified nomenclature should not only encompass the complex clinical and pathological features associated with *RYR1* variations (including conditions that do not exhibit an overt myopathy such as MH susceptibility and exertional rhabdomyolysis), but also both accommodate future *RYR1*-related phenotypes and navigate the field away from utilizing non-specific histopathologic eponyms (Fig. 3).

## Conclusion

Historically, *RYR1*-RD have been named and diagnosed based largely on histopathologic findings on muscle biopsy. However, the emergence of new subtypes along the *RYR1* disease spectrum complicates diagnoses. As a “genetics-first” approach to inherited disease diagnosis is becoming widely accepted, neuromuscular disorders such as *RYR1*-RD need established guidelines and consensus principles for classification and naming of emerging phenotypes. The proposal for *RYR1*-RD as the unifying nomenclature is a first step and could be superceded by a better encompassing terminology. We believe that such a discourse is timely and needed for this widely heterogeneous group of muscle disorders.

## Data Availability

Data sharing is not applicable to this article as no datasets were generated or analyzed.

## Abbreviations

CM: Congenital myopathies
Ca^2+^: Calcium
CCD: Central core disease
CFTD: Congenital fiber-type disproportion
CNM: Centronuclear myopathy
CNMDU1: Congenital neuromuscular disease with uniform type 1 fiber
EC: Excitation-contraction
MH: Malignant hyperthermia
MmD: Multi-minicore disease
RYR1: Ryanodine receptor type 1
RYR1-RM: Ryanodine receptor type 1-related myopathies
SR: Sarcoplasmic reticulum
VUS: Variant of unknown significance
